# Role of postoperative radiotherapy in dermatofibrosarcoma protuberans: a propensity score-matched analysis

**DOI:** 10.1186/s13014-019-1226-z

**Published:** 2019-01-29

**Authors:** Kaixin Du, Jinluan Li, Lirui Tang, Xiaoyi Lin, Xiangquan Kong, Xuehong Liao, Qingqin Peng, Yaping Dong, Junyan He, Yunxia Huang, Xueqing Zhang, Feifei Lin, Qingyang Zhuang, Junxin Wu

**Affiliations:** 1 Department of Radiation Oncology, Xiamen Humanity Hospital, Xiamen, 361000 China; 2 Department of Radiation Oncology, Fujian Medical University Cancer Hospital, Fujian Cancer Hospital, Fuzhou, 350014 China; 3 Department of Pathology, Xiamen University Affiliated Zhongshan Hospital, Xiamen, China; 4 Department of Oncology, Graduate School of Fujian Medical University, Fuzhou, China; 5 Department of Biochemistry and Biology, University of South, Hengyang, China

**Keywords:** DFSP, Ki-67, PSM, Radiation

## Abstract

**Objective:**

This study aimed to evaluate the role of postoperative radiotherapy (RT) in dermatofibrosarcoma protuberans (DFSP) and identify the prognostic factors influencing the disease-free survival (DFS).

**Methods:**

A total of 184 patients with DFSP were analyzed from 2000 to 2016. The regression model was used to examine the prognostic factors for DFS. Baseline covariates were balanced using a propensity score model. The role of RT was assessed by comparing the DFS of the surgery + RT group with that of the surgery group.

**Results:**

The median follow-up was 58 months (range, 6–203 months). The 5-year DFS rate was 89.8%. The univariate analysis showed that age ≥ 50 years, presence of fibrosarcoma, margins < 2 cm, and tumor size ≥5 cm were associated with worse DFS (*P* = 0.002, *P* <  0.001, *P* = 0.030, and *P* = 0.032, respectively). The multivariate Cox regression model revealed that age, margin width, lesion number, and histological subtype independently affected DFS. The Ki-67 expression was related to age and histological subtype. Patients with Ki-67 ≥ 17% showed a worse DFS than those with Ki-67 < 17% (35.8% vs 87.8%, *P* = 0.002). In the matched cohort, DFS was significantly higher in the S + RT group than in the S group (5-year DFS, 88.1% vs 56.2%, *P* = 0.044).

**Conclusions:**

Age, margin width, lesion number, and histological subtype were independent risk factors for DFS in patients with DFSP. The high expression of Ki-67 could predict a poor prognosis. Postoperative RT could improve DFS for patients with DFSP.

## Introduction

Dermatofibrosarcoma protuberans (DFSP) is a rare skin tumor that accounts for < 0.1% of all cancers and approximately 1% of all soft tissue sarcomas [1, 2]. It is a low-grade malignancy that rarely metastasizes. It is characterized by locally invasive infiltration, and a local resection results in a high recurrence rate of up to 60% [3]. Hence, the goal of the therapy for DFSP is to reach a wide and clear resection margin of 2–3 cm to reduce the local recurrence rate [4, 5]. On the contrary, approximately 5–15% of all cases undergo fibrosarcomatous changes (FS-DFSP), which can increase the risk of recurrence and metastasis [6, 7]. Nevertheless, the significance of adjuvant radiotherapy (RT) in DFSP remains controversial [8].

Markers to predict the prognosis of DFSP in clinical practice are still lacking. Ki-67, which is indispensable in cell proliferation, is related to the occurrence, development, metastasis, and prognosis of a tumor [9]. However, the role of Ki-67 as a prognostic factor in DFSP is not yet clarified.

Hitherto, no consensus on the prognostic factors of DFSP has been achieved. Thus, the present study aimed to find appropriate prognostic indicators to identify high-risk patients and evaluate the role of RT using a propensity score-matched (PSM) analysis.

## Materials and methods

### Patients

This study was approved by the Ethics Committee of the Fujian Cancer Hospital (No. KT2016–012-01) and was performed in accordance with the Helsinki Declaration. A total of 184 patients with DFSP consecutively treated at the Fujian Cancer Hospital from January 2000 to 2016 were retrospectively analyzed. The pretreatment evaluation included complete history, physical examination, and laboratory tests of patients. The inclusion criteria were as follows: (1) pathologically confirmed DFSP; (2) Karnofsky performance status > 70; (3) no previous or concurrent malignancy; and (4) no evidence of distant metastasis.

### Treatment

The surgical approach and procedure were determined based on tumor location and preference of surgeon. All patients underwent resection, and the width of surgical margins depended on the tumor location. RT was delivered within a month after surgery. The patients were treated with 6-MV photon beams alone or in combination with electron boost fields. RT doses were delivered at 2 Gy/fraction and 5 fractions/week. The size of the radiation field was determined by the location and size of tumor and the placement of surgical scar. Of the 44 patients, 22 were treated with 50 Gy/25 fractions, 17 with 60 Gy/30 fractions, and 5 with 66 Gy/33 fractions. RT doses were 50 Gy/25 fractions to the tumor bed extended by 3–5 cm and with/without 10–16 Gy electron boost to the tumor bed extended by 1 cm for patients with positive or insufficient margins.

### Follow-up

All patients were clinically followed up every 3 months for the first 2 years, every 6 months for an additional 3 years, and then annually for the rest of life. Local progression was defined as local recurrence in the previously treated region.

### Statistical analysis

Variables were screened by univariate analysis, and clinically significant factors were incorporated into the Cox regression model to examine the prognostic factors for DFS. The cutoff point of Ki-67 expression affecting DFS was determined by the Cutoff Finder application [6]. The correlations between Ki-67 expression and clinical factors were compared using the chi-square test or Fisher’s exact test. The propensity score matching ratio was set to 1:1 to minimize the differences in clinicopathological factors, and two well-balanced groups were created. Survival curves were constructed using the Kaplan–Meier method and compared using the log-rank test. Statistical analyses were performed using the SPSS version 22.0 (IBM Corporation, NY, USA). All tests of significance were two sided, and differences with a *P* value < 0.05 were significant.

## Results

### Patient characteristics

A total of 184 patients [140 (76.1%) male and 44 (23.9%) female] were included in the analysis. The characteristics of these patients are shown in Table 1. The median age was 41 (range, 8–82) years. The trunk (71.7%, 132/184) was the most common site involved, followed by the head and neck (17.4%, 32/184), and extremities (10.9%, 20/184). The median tumor size was 3 (range, 1–20) cm. Among these 184 patients, the margin was < 2 cm in 47 (25.5%) patients and ≥ 2 cm in 137 (74.5%) patients. Moreover, the cohort consisted of 161 patients (87.5%) of ordinary DFSP, 16 (8.7%) of fibrosarcoma DFSP, and 7 (3.8%) of myxoid DFSP. A total of 44/184 (23.9%) patients received RT. Further, 37/56 patients (66.1%) had the low expression of Ki-67, and 19/56 patients (33.9%) showed high expression.

**Table 1:** Patients’ characteristics (*n* = 184)

Characteristic	n (%)
Presentation	
Primary	122 (66.3)
Recurrent	62 (33.7)
Gender	
Male	140 (76.1)
Female	44 (23.9)
Age(years)	
< 50	129 (70.1)
≥ 50	55 (29.9)
Site	
Trunk	132 (71.7)
Extremities	20 (10.9)
Head and neck	32 (17.4)
Tumor size, cm	
< 5	133 (72.3)
≥ 5	51 (27.7)
Lesion number	
1	171 (92.9)
≥ 2	13 (7.1)
Margin status	
Negative	170 (92.4)
Positive	14 (7.6)
Margin width, cm	
< 2	47 (25.5)
≥ 2	137 (74.5)
Radiotherapy	
No	140 (76.1)
Yes	44 (23.9)
Histological subtype	
Ordinary	161 (87.5)
Fibrosarcoma	16 (8.7)
Myxoid	7 (3.8)
Ki-67 (%)	
< 17	37 (66.1)
≥ 17	19 (33.9)

### Overall survival and disease-free survival for the overall sample

The median follow-up time was 58 (range, 6–203) months. The median disease-free survival (DFS) time was 55 (range, 6–197) months. A total of 17/184 (15.5%) patients experienced progression; 6/184 (3.2%) were found to have distant metastases. The 3- and 5-year overall survival (OS) in patients was 98.8% [95% confidence interval (CI), 97.0–100] and 95.7% (95% CI: 91.9–99.4), respectively. The 3- and 5-year DFS was 94.6% (95% CI: 91.2–97.9) and 89.8% (95% CI: 84.5–95.1), respectively.

### Prognostic factors affecting DFS

**Fig. 1:**
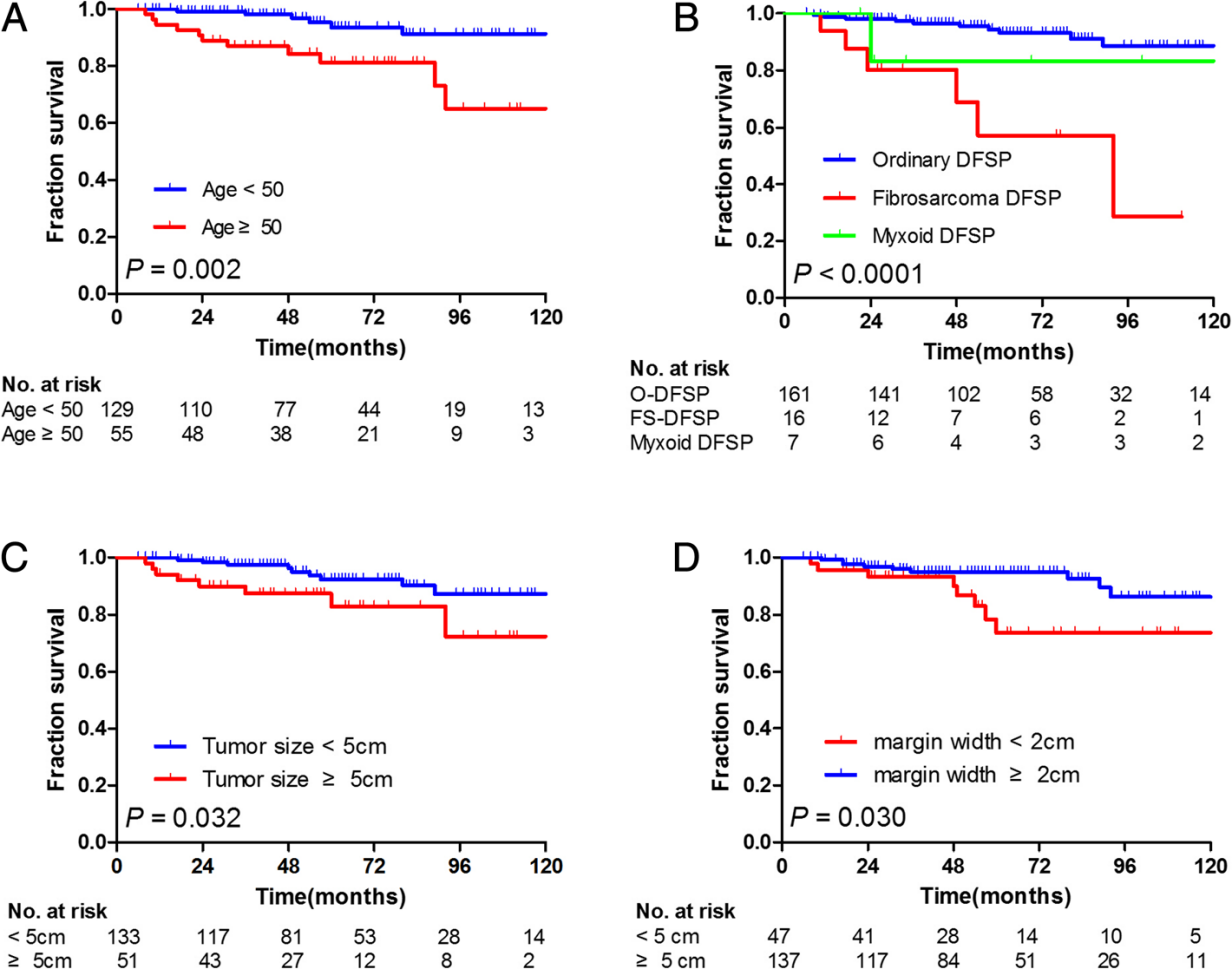
Kaplan–Meier analysis of DFS according to (**a**) age (50 vs < 50), (**b**) histological subtype (ordinary, fibrosarcoma, and myxoid), and (**c**) tumor size (< 5 cm vs ≥5 cm), and (**d**) margin width (< 2 cm vs ≥2 cm)

The univariate and multivariate analyses of factors influencing DFS are summarized in Table 2. The univariate analysis showed that age ≥ 50 years, presence of fibrosarcoma, margins < 2 cm, and tumor size ≥5 cm were significantly associated with DFS (*P* = 0.002, *P* <  0.001, *P* = 0.030, and *P* = 0.032, respectively). The multivariate Cox regression model revealed that age ≥ 50 years (*P* = 0.003), margins < 2 cm (*P* = 0.006), lesion number (*P* = 0.026), and histological subtype (*P* = 0.043) significantly affected DFS. These survival curves are shown in Fig. 1. The lesion number tended to correlate with DFS (*P* = 0.072). Of these variables, age [*P* = 0.003, hazard ratio (HR) = 4.699, 95% CI: 1.672–13.204], margin width (*P* = 0.006, HR = 0.211, 95% CI: 0.070–0.633), lesion number (*P* = 0.026, HR = 0.199, 95% CI: 0.048–0.820), and histological subtype (*P* = 0.043, HR = 1.645, 95% CI: 1.016–2.665) were independently associated with DFS.

**Table 2:** Univariate and multivariate analysis of disease-free survival

	Univariate		Multivariate		
Variables	5-year DFS (95% CI)	*P* value (log-rank) *	Hazard ratio	95% CI	*P* value
Presentation		0.253			
Primary	92.4 (92.3–92.5)				
Recurrent	85.4 (85.3–85.5)				
Gender		0.151			
Male	87.8 (87.7–87.9)				
Female	95.3 (95.2–95.4)				
Age(years)		0.002	4.699	1.672–13.204	0.003
< 50	93.7 (93.6–93.8)				
≥ 50	81.3 (81.2–81.4)				
Site		0.296			
Trunk	90.3 (90.2–90.4)				
Extremities	92.3 (92.2–92.4)				
Head and neck	85.9 (85.8–86.0)				
Tumor size, cm		0.032	2.267	0.844–6.091	0.105
< 5	92.4 (92.3–92.5)				
≥ 5	82.8 (82.7–82.9)				
Lesion number		0.072	0.199	0.048–0.820	0.026
1	90.3 (90.2–90.4)				
≥ 2	84.6 (84.4–84.8)				
Margin status		0.121			
Negative	91.5 (86.2–96.8)				
Positive	80.0 (61.0–99.0)				
Margin width, cm		0.030	0.211	0.070–0.633	0.006
< 2	73.7 (73.5–73.9)				
≥ 2	95.1 (95.1–95.1)				
Radiotherapy		0.171			
No	92.5 (92.4–92.6)				
Yes	82.8 (82.7–82.9)				
Histological subtype		< 0.001	1.645	1.016–2.665	0.043
Ordinary	93.2 (93.2–93.2)				
Fibrosarcoma	57.3 (57.0–57.3)				
Myxoid	83.3 (83.0–83.6)				

DFS: disease-free survival, CI confidence interval. *Log-rank test for equality of survivor functions. **Lesion number at the original site

### Correlation between Ki-67 expression and clinicopathological characteristics

**Fig. 2:**
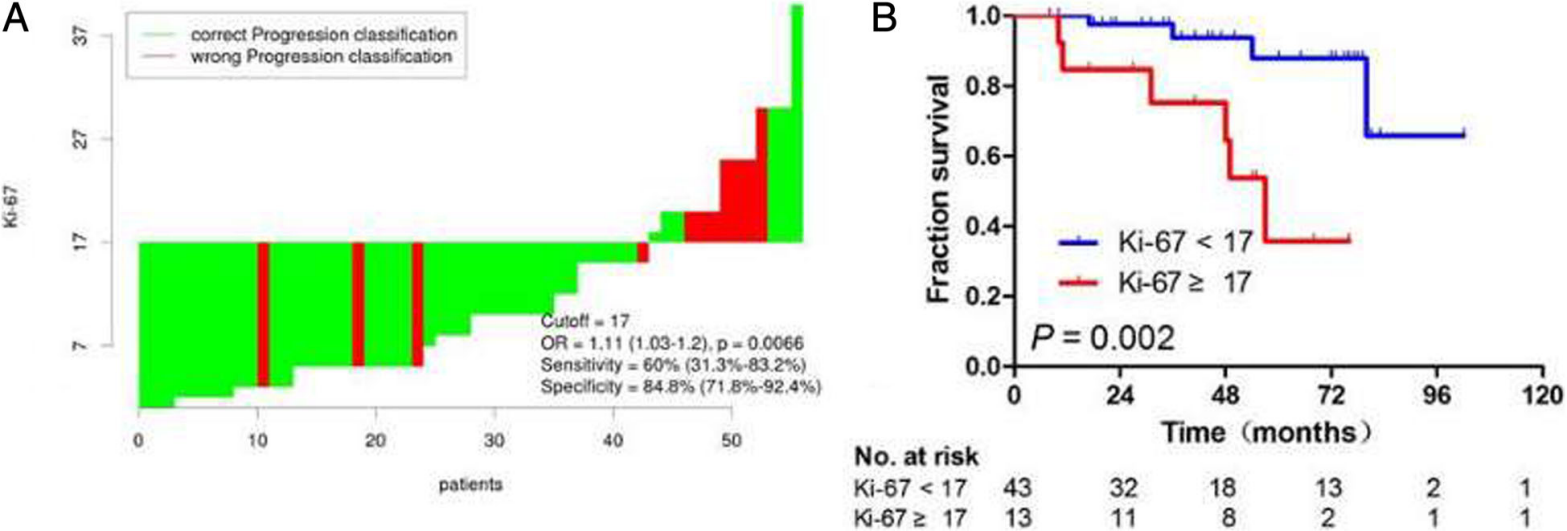
Cutoff optimization of Ki-67 by correlation with DFS in the DFSP data. **a** The hazard ratio (HR) including 95% CI is plotted depending on the cutoff. A vertical line designates the dichotomization showing the most significant correlation with DFS. The distribution of Ki-67 in the 56 tumors is shown as a rug plot at the bottom of the figures. **b** Kaplan–Meier analysis of DFS for Ki-67 < 17% and Ki-67 ≥ 17%

The results of cutoff point determination for Ki-67 indicated that 17% was the optimal point, which was supported by the ROC method of Cutoff Finder. The specificity was 60% (31.3–83.2%) and the sensitivity 84.8% (71.8–92.4%) (Fig. 2a). The area under the curve was 0.73 (*P* = 0.023, 95% CI: 0.542–0.921). Compared with high Ki-67 expression, the low expression showed a significantly high 5-year DFS in patients with DFSP (87.8 vs 35.8%, *P* = 0.002), as shown in Fig. 2b. The comparison of clinicopathological characteristics between the different levels of Ki-67 expression is shown in Table 3. The Ki-67 expression was associated with age (*P* = 0.047) and histological subtype (*P* = 0.003). The differences in presentation, gender, site, tumor size, lesion number, margin status, and margin width between the two groups were not statistically significant (*P* > 0.05).

**Table 3:** Comparison of clinicopathologic characteristics between low and high Ki-67 expression

Characteristic	n (%)	Ki-67 < 17%	Ki-67 ≥ 17%	*P*
Presentation				0.942
Primary	35(62.5)	23(62.2)	12(63.2)	
Recurrent	21(37.5)	14(37.8)	7(36.8)	
Gender				0.749
Male	49(87.5)	32(86.5)	17(89.5)	
Female	7(12.5)	5(13.5)	2(10.5)	
Age(years)				0.047
< 50	39(69.6)	29(78.4)	10(52.6)	
≥ 50	17(30.4)	8(21.6)	9(47.4)	
Site				0.465
Trunk	41(73.2)	29(78.4)	12 (63.2)	
Extremities	6(10.7)	3(8.1)	3 (15.8)	
Head and neck	9(16.1)	5(13.5)	4 (21.1)	
Tumor size, cm				0.192
< 5	36(64.3)	26(70.3)	10(52.6)	
≥ 5	20(35.7)	11(29.7)	9(47.4)	
Lesion number				0.749
1	49(87.5)	32(86.5)	17(89.5)	
≥ 2	7(12.5)	5(13.5)	2(10.5)	
Margin status				0.961
Negative	44(78.6)	29(78.4)	15(78.9)	
Positive	12(21.4)	8(21.6)	4(21.1)	
Margin width, cm				0.562
< 2	15(26.8)	9(24.3)	6(31.6)	
≥ 2	41(73.2)	28(75.7)	13(68.4)	
Histological subtype				0.003
Ordinary	45(80.4)	34(91.9)	11(57.9)	
Fibrosarcoma	10(17.9)	2(5.4)	8(42.1)	
Myxoid	1(1.7)	1(2.7)	0(0.0)	

### PSM cohort

**Fig. 3:**
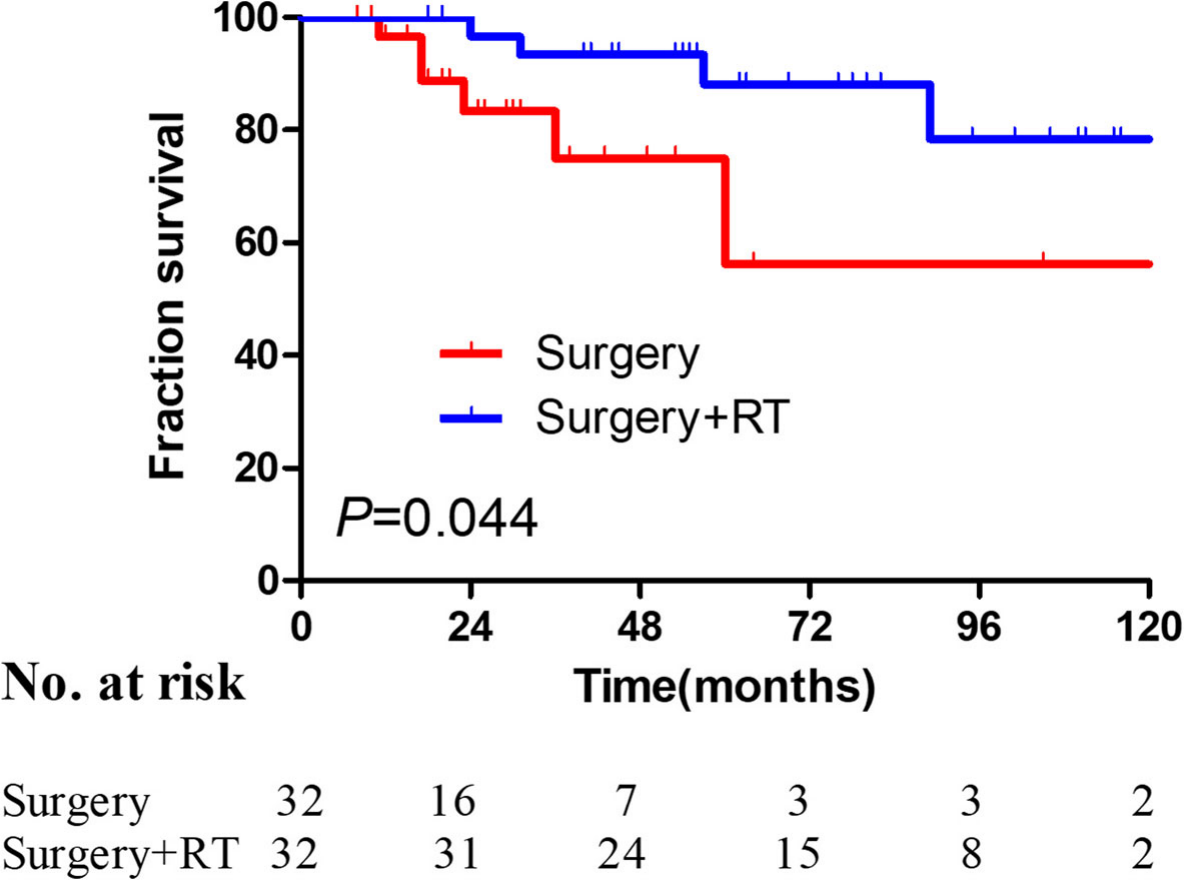
Kaplan–Meier analysis of DFS for the surgery and surgery + RT groups after matching (5-year DFS: 56.2% vs 88.1%, *P* = 0.044)

Thirty-two pairs of patients from the S + RT and S groups were matched one-to-one using PSM. The clinical characteristics, including age, margin width, lesion number, and histological subtype, of the two groups did not differ significantly after the PSM (Table 4). Among the matched samples, the S + RT group had longer 5-year DFS compared with the S group (88.1% vs 56.2%, *P* = 0.044, Fig. 3).

**Table 4:** Patients’ characteristics before and after Propensity Score Matching

Characteristics	Before Matching					After Matching				
	S		S + RT		*P*	S		S + RT		*P*
	*n* = 140	%	*n* = 44	%		*n* = 32	%	*n* = 32	%	
Gender					0.315					0.396
Male	109	77.9	31	70.5		25	78.1	22	68.8	
Female	31	22.1	13	29.5		7	21.9	10	31.3	
Presentation					0.949					1.000
Primary	93	66.4	29	65.9		21	65.6	21	65.6	
Recurrent	47	33.6	15	34.1		11	34.4	11	34.4	
Age, years					0.234					1.000
< 50	95	67.9	34	77.3		24	75.0	24	75.0	
≥ 50	45	32.1	10	22.7		8	25.0	8	25.0	
Site					0.214					0.815
Trunk	100	71.4	32	72.7		24	75.0	24	75.0	
Extremities	18	12.9	2	4.6		2	6.2	1	3.1	
Head and neck	22	15.7	10	22.7		6	18.8	7	21.9	
Tumor size, cm					0.397					0.784
< 5	99	70.7	34	77.3		22	68.8	23	71.9	
≥ 5	41	29.3	10	22.7		10	31.2	9	28.1	
Lesion number					0.455					0.301
1	129	92.1	42	95.5		29	90.6	31	96 .9	
≥ 2	11	7.9	2	4.5		3	9.4	1	3.1	
Margin status					< 0.001					0.391
Negative	126	90.0	27	61.4		30	93.8	28	87.5	
Positive	14	10.0	17	38.6		2	6.2	4	12.5	
Margin width, cm					< 0.001					0.617
< 2	19	13.6	28	63.6		15	46.9	17	53.1	
≥ 2	121	86.4	16	36.4		17	53.1	15	46.9	
Histological					0.183					0.586
Ordinary	124	87.9	37	84.1		28	88.6	30	82.8	
Fibrosarcoma	13	9.3	3	6.8		3	8.6	1	8.6	
Myxoid	3	2.1	4	9.1		1	2.9	1	8.6	

*S* surgery, *RT* radiotherapy

## Discussion

The present study provided one of the largest cohorts for DFSP, a rare cutaneous tumor with good prognosis. The long-term follow-up revealed that 5-year DFS and OS were 89.8% (95% CI: 84.5–95.1) and 95.7% (95% CI: 92.0–99.4), respectively. The multivariate analysis showed that age, margin width, lesion number, and histological subtype were the independent prognostic factors affecting DFS. This novel study proposed Ki-67 as a prognostic molecular marker in patients with DFSP. The PSM analysis revealed that postoperative RT improved DFS in these patients.

The safety margin of DFSP resection is the hotspot because infiltrating growth is its major feature. An adequate surgical margin remains the key to reduce the recurrence of DFSP. Several published studies recommended that surgical margin width should be 2–4 cm [10–12]. The results of the present study also demonstrated that the margin width ≥ 2 cm was the independent prognostic factor for DFS (HR = 0.124, 95% CI: 0.020–0.763, *P* = 0.024). In this study, patients with margin width <  2 cm had significantly worse DFS compared with patients with margin width ≥ 2 cm (*P* = 0.030). Reimann et al. and Han et al. also reported that DFSP with inadequate surgical margins had poor outcomes [13, 14].

DFSP is divided into four histologic types: ordinary, fibrosarcoma, pigmented, and myxoid. A total of 16 patients with FS-DFSP were enrolled for this study. Of these, five (31.3%) had a local recurrence, and four (28.6%) had a metastasis. Patients with FS-DFSP presented with worse DFS compared with other types (93.2, 83.3, and 57.3% after 5 years for ordinary, myxoid, and fibrosarcoma, respectively), which was in agreement with previous reports [15, 16]. Limited data are available on myxoid DFSP because it is extremely rare. A study with a small sample of myxoid DFSP reported two cases (25%) with local recurrence and no metastasis [17]. The present study included seven cases of myxoid DFSP, and none of them displayed any local recurrence. Nevertheless, one case was found to have lung metastasis within 2 years after surgery and died 2 years after the metastasis. Considering the poor prognosis of these two types, wide resection margins (≥2 cm) should be recommended, especially in FS-DFSP.

No report described the effect of lesion number on DFS in DFSP. The univariate analysis revealed the correlation of the lesion number with DFS (*P* = 0.072, 5-year DFS: 90.3% vs 84.6%). Moreover, it was a significantly independent prognostic factor as evaluated by multivariate analysis (*P* = 0.025). It was speculated that the lesion number might affect the margin of resection and indirectly affect DFS in DFSP. Bowne et al. [17] and Giovanni et al. [18] reported that age ≥ 50 years was an unfavorable prognostic factor for DFSP. However, Gayner et al. found no significant correlation between DFS and age of patients [19]. The results showed that age ≥ 50 years was an independent adverse prognostic factor as revealed by the multivariate analysis. The 5-year DFS was significantly lower in elderly patients than in those aged < 50 years (81.3 vs 93.7%, *P* = 0.002). However, the mechanism is yet elusive.

Ki-67 is a protein involved in cell proliferation and a reliable indicator for detecting tumor proliferation activity. Previous studies reported that the expression of Ki-67 was related to metastasis and prognosis of non–small cell lung cancer and colon cancer [3, 15]. Besides Ki-67, other indicators have already been documented as potential prognostic variables in DFSP [2]. According to the univariate analysis, patients with high Ki-67 expression had poorer 5-year DFS compared with others (47.6 vs 88.8%, *P* = 0.002). Three patients having distant metastases were detected for Ki-67 expression, and two (66.7%) showed high Ki-67 expression. Thus, the present study indicated that Ki-67 might also be used as a prognostic factor for DFSP. It is recommended to detect Ki-67 for patients with DFSP in terms of prognostic evaluation, lest the predictive value of Ki-67 for DFSP is usually overlooked clinically. However, expanding the sample size to further confirm the preliminary result of this study seems necessary due to the limitation of insufficient Ki- 67 detection (only 56 of 184 DFSP).

The local recurrence rate was as high as 60% after the inadequate excision of DFSP, and it decreased after enlarged resection (7.3%) [20]. Although a sufficient margin is the key to reduce the local recurrence rate, it is often limited by the anatomical site. Hence, the present study focused on the adjuvant therapy to reduce the local recurrence. RT is a noninvasive treatment that can improve local control in patients having close or positive margins [21–23]. The results published by Tsai et al. suggested that DFSP could benefit from postoperative RT [16]. Recently, a meta-analysis reported that patients undergoing postoperative RT had a lower recurrence rate compared with those undergoing surgery alone [4]. However, Huber et al. speculated that the effect of postoperative RT was limited in patients with DFSP [24]. After using PSM to minimize the selection bias between S and S + RT groups, two groups of patients exhibited a reduced effect of potential confounding factors, and patients’ backgrounds were adjusted for a similar outcome. Furthermore, the results indicated that the 5-year DFS of the S + RT group was better than that of the S group (88.1 vs 56.2%, *P* = 0.044). Thus, the present study concluded that postoperative RT could improve DFS in patients with DFSP.

Nevertheless, this retrospective single-center study had several limitations. First, further studies on the application of Ki-67 are essential because Ki-67 testing was not widely applied in DFSP in this study. Second, although this was one of the largest cohorts of DFSP, the sample size was limited after PSM.

In conclusion, this study found that age, margin width, lesion number, and histological subtype were the independent risk factors for DFS in patients with DFSP. Also, postoperative RT could substantially improve DFS for high-risk DFSP. Further, this novel study indicated that Ki-67 might become a prognostic molecular marker in patients with DFSP.

## Abbreviations

DFS: Disease-free survival
DFSP: Dermatofibrosarcoma protuberans
RT: Radiotherapy

## References

[1] DW Buck II, Kim J, Alam M (2012). Multidisciplinary approach to the management of dermatofibrosarcoma protuberans. J Am Acad Dermatol.

[2] Tsai YJ, Lin PY, Chew KY, Chiang YC (2014). Dermatofibrosarcoma protuberans in children and adolescents: clinical presentation, histology, treatment, and review of the literature. J Plast Reconstr Aesthet Surg.

[3] Llombart B, Serra-Guillén C, Monteagudo C, López Guerrero JA, Sanmartín O (2013). Dermatofibrosarcoma protuberans: a comprehensive review and update on diagnosis and management. Semin Diagn Pathol.

[4] Chen YT, Tu WT, Lee WR, Huang YC (2016). The efficacy of adjuvant radiotherapy in dermatofibrosarcoma protuberans: a systemic review and meta-analysis. J. Eur. Acad. Dermatol. Venereol.

[5] Liang CA, Jambusaria-Pahlajani A, Karia PS, Elenitsas R, Zhang PD, Schmults CD (2014). A systematic review of outcome data for dermatofibrosarcoma protuberans with and without fibrosarcomatous change. J Am Acad Dermatol.

[6] Wu SG, Wang Y, Zhou J, Sun JY, Li FY, Lin HX (2015). Number of negative lymph nodes should be considered for incorporation into staging for breast cancer. Am J Cancer Res.

[7] Chan TC, Wu CJ, Jeng SF (2015). Dermatofibrosarcoma protuberans: a 10-year experience. Formosan J Surg.

[8] Hanprasertpong J, Tungsinmunkong K, Chichareon S, Wootipoom V, Geater A, Buhachat R, et al. Correlation of p53 and Ki (MIB expressions with clinicopathological features and prognosis of early stage cervical squamous cell carcinomas. Journal of Obstetrics & Gynaecology Research. 2010;36:572-80 10.1111/j.1447-0756.2010.01227.x 20598040

[9] Cai H, Wang Y, Wu J, Shi Y (2012). Dermatofibrosarcoma protuberans: clinical diagnoses and treatment results of 260 cases in China. J Surg Oncol.

[10] Dubay D, Cimmino V, Lowe L, Johnson TM, Sondak VK (2010). Low recurrence rate after surgery for dermatofibrosarcoma protuberans: a multidisciplinary approach from a single institution. Cancer.

[11] Stojadinovic A, Karpoff HM, Antonescu CR, Shah JP, Singh B, Spiro RH (2000). Dermatofibrosarcoma protuberans of the head and neck. Ann Surg Oncol.

[12] Farma JM, Ammori JB, Zager JS, Marzban SS, Bui MM, Bichakjian CK (2010). Dermatofibrosarcoma Protuberans: how wide should we resect?. Ann Surg Oncol.

[13] Reimann JD, Fletcher CD (2007). Myxoid dermatofibrosarcoma protuberans: a rare variant analyzed in a series of 23 cases. Am J Surg Pathol.

[14] Han B, Lin S, Yu LJ, Wang RZ, Wang YY (2009). Correlation of ^18^F-FDG PET activity with expressions of survivin, Ki67, and CD34 in non-small-cell lung cancer. Nucl Med Commun.

[15] Ma YL, Peng JY, Zhang P, Liu WJ, Huang L, Qin HL (2010). Immunohistochemical analysis revealed CD34 and Ki67 protein expression as significant prognostic factors in colorectal cancer. Med Oncol.

[16] Tsai CJ, Castle KO, Guadagnolo B, Feig BW, Zagars GK (2012). Dermatofibrosarcoma Protuberans: Long-term outcomes of 53 patients treated with conservative surgery and radiation therapy. Int J Radiat Oncol Biol Phys.

[17] Bowne WB, Antonescu CR, Leung DH, Katz SC, Hawkins WG, Woodruff JM (2000). Dermatofibrosarcoma protuberans: a clinicopathologic analysis of patients treated and followed at a single institution. Cancer.

[18] Paolino G, Didona D, Bottoni U, Romaniello F, Corsetti P, Richetta AG (2017). Dermatofibrosarcoma protuberans: when the age makes the difference.

[19] Gayner SM, Lewis JE, Mccaffrey TV (1997). Effect of resection margins on dermatofibrosarcoma protuberans of the head and neck. Arch Otolaryngol Head Neck Surg.

[20] Akram J, Wooler G, Lockandersen J (2014). Dermatofibrosarcoma protuberans: clinical series, national Danish incidence data and suggested guidelines. Scand. J. Plast. Reconstr. Surg.

[21] Dagan R, Morris CG, Zlotecki RA, Scarborough MT, Mendenhall WM (2005). Radiotherapy in the treatment of dermatofibrosarcoma protuberans. Am J Clin Oncol.

[22] Ballo MT, Zagars GK, Pisters P, Pollack A (1998). The role of radiation therapy in the management of dermatofibrosarcoma protuberans. Int. J. Radiat. Oncol. Biol. Phys.

[23] Sun LM, Wang CJ, Huang CC, Leung SW, Chen HC, Fang FM (2000). Dermatofibrosarcoma protuberans: treatment results of 35 cases. Radiother Oncol.

[24] Huber GF, Matthews TW, Dort JC (2007). Radiation-induced soft tissue sarcomas of the head and neck. J Otolaryngol.

